# The Use of Vasoactive Drugs in the Treatment of Male Erectile Dysfunction: Current Concepts

**DOI:** 10.3390/jcm9092987

**Published:** 2020-09-16

**Authors:** George T. Kedia, Stefan Ückert, Dimitrios Tsikas, Armin J. Becker, Markus A. Kuczyk, Andreas Bannowsky

**Affiliations:** 1 Department of Urology & Urological Oncology, Division of Surgery, Hannover Medical School, 30625 Hannover, Germany; georgekedia@yahoo.de (G.T.K.); streetgang@gmx.de (S.Ü.); 2 Department of Urology, DIAKOVERE GmbH, Friederikenstft Lutheran Hospital, 30171 Hannover, Germany; 3 Center of Pharmacology & Toxicology, Core Unit Proteomics, Hannover Medical School, 30625 Hannover, Germany; Tsikas.Dimitros@mh-hannover.de; 4 Faculty of Medicine, Academic Hospital Grosshadern, Department of Urology, Ludwig Maximilians University, 81377 Munich, Germany; Armin.Becker@med.uni-muenchen.de; 5 Imland Hospital, Department of Urology, 24768 Rendsburg, Germany; andreas.bannowsky@imland.de

**Keywords:** erectile dysfunction (ED), drug treatment, vasoactive compounds, nitric oxide (NO)/cyclic guanosine monophosphate (cyclic GMP), cyclic adenosine monophosphate (cyclic AMP)

## Abstract

It is widely accepted that disorders of the male (uro)genital tract, such as erectile dysfunction (ED) and benign diseases of the prostate (lower urinary tract symptomatology or benign prostatic hyperplasia), can be approached therapeutically by influencing the function of both the vascular and non-vascular smooth muscle of the penile erectile tissue or the transition zone/periurethral region of the prostate, respectively. As a result of the discovery of nitric oxide (NO) and cyclic guanosine monophosphate (GMP) as central mediators of penile smooth muscle relaxation, the use of drugs known to increase the local production of NO and/or elevate the intracellular level of the second messenger cyclic GMP have attracted broad attention in the treatment of ED of various etiologies. Specifically, the introduction of vasoactive drugs, including orally active inhibitors of the cyclic GMP-specific phosphodiesterase (PDE) 5, has offered great advantage in the pharmacotherapy of ED and other diseases of the genitourinary tract. These drugs have been proven efficacious with a fast on-set of action and an improved profile of side-effects. This review summarizes current strategies for the treatment of ED utilizing the application of vasoactive drugs via the oral, transurethral, topical, or self-injection route.

## 1. Introduction

Erectile dysfunction (ED) is defined as the consistent inability to attain or maintain penile erection sufficient for conducting sexual intercourse [1]. According to data from worldwide epidemiological surveys, the prevalence of ED is 20–40% at 40 years of age and an overall 70% in the decade from 70 years to 80 years. It is estimated that approximately 150 million men suffer from ED [2,3]. The vascular system provides adequate blood supply to the corpora cavernosa of the penis, thus facilitating the so-called veno-occlusive mechanism required for inducing and maintaining an erection. Since pathological alterations of the vascular system can significantly compromise erectile function, diseases such as atherosclerosis/vascular ischemia, hypertension, lipidemia/hypercholesterolemia, diabetes mellitus (known to promote peripheral neuropathies and, eventually, vascular deteriorations) and generalized endothelial dysfunction can cause erectile dysfunction. ED has been shown to be associated with an increased risk of developing severe coronary heart disease or a stroke event within a time interval of 10 years [4]. Thus, there is a high prevalence of ED in patients sharing risk factors known to contribute to the on-set of endothelial dysfunction, characterized by an impairment in the endothelium-dependent vasodilation triggered by NO (FMD = flow-mediated vasodilation brought about by sheer stress) [5,6,7]. To date, extensive coverage has been made on the NO/cyclic guanosine monophosphate (cGMP) pathway in penile erectile tissue [8]. This landmark discovery has enabled the development of drugs enhancing the intracellular levels of cyclic GMP, in particular, the phosphodiesterase (PDE)5 inhibitors sildenafil, tadalafil, vardenafil and avanafil. In addition, compounds acting via cyclic adenosine monophosphate (cyclic AMP), such as prostaglandin E1 (PGE1) and vasoactive intestinal polypeptide (VIP), are used in self-injection or topical regimens for the management of ED [9,10]. Via binding to specific receptors located in the outer membrane, said compounds can stimulate the activity of the cyclic AMP producing enzyme adenylyl cyclase (AC). Cyclic AMP stimulates the activity of cyclic AMP-dependent protein kinases which, in turn, interact with Ca^2+^-channels, thereby antagonising contraction responses of smooth muscle cells. In human corpus cavernosum tissue, the intracellular level of cyclic AMP is under the control of the cyclic AMP-degrading PDE isoenzymes type 3 and 4 [11].

## 2. Recent PDE5 Inhibitors in the Treatment of Erectile Dysfunction

Inhibitors of PDE5 are characterized as non-hydrolysable analogues to the second messenger molecule cyclic GMP. PDE5 inhibitors competitively bind to the catalytic site of the enzyme, thus counteracting the degradation of cyclic GMP, subsequently enhancing the relaxation response of the penile erectile tissue brought about by NO. Sildenafil and vardenafil share some major structural domains: a nucleobase (guanine)-like structure (presenting a keto-group and a non-cyclic side chain), a ribose, and a pentacyclic domain coupled to the sugar residue via an amine, sulphonamide/sulphur dioxide residue, preventing the molecule from hydrolytic degradation by the PDE5. In the tadalafil molecule, the ribose residue has been substituted by a phenyl group, and the sulphonamide ligand replaced by an oxygen-containing bicyclic structure. This results in little activity of the tadalafil molecule against other PDE isoenzymes. Avanafil is significantly different from the standard model described above. This property enables avanafil to bind to the catalytic site of the PDE5 regardless of the spatial orientation of the inhibitor molecule and, thus, increases its affinity towards the PDE5 [12]. Sildenafil, vardenafil and avanafil are short acting agents, while tadalafil has a longer pharmacological T_lambda_, thereby allowing the user greater flexibility in performing sexual activity. An unimpaired neuronal innervation of the erectile tissue as well as intact cavernous structures, providing for the production of sufficient amounts of NO from (vascular) endothelial or neuronal sources, are pivotal for the pharmacological action of PDE5 inhibitors [13]. The clinical efficacy of PDE5 inhibitors has been evaluated comprehensively in various cohorts of male patients with different causes of ED (psychogenic, due to diabetes, cardiovascular diseases or conditions causing damage to the pudendal or cavernous nerves, such as spinal cord injuries or histories of pelvic surgeries, for example, cystectomy or prostatectomy). In all trials, the PDE5 inhibitors were superior when compared to placebo; overall response rates were calculated at 60–80% [14]. In general, the clinical efficacy outcome increased with escalation of the dose of the PDE5 inhibitors. Favorable response rates (46–72%) have also been reported in cohorts of patients with ED following uni- or bilateral nerve sparing radical prostatectomy (NSRP). In various randomized, multicenter, prospective trials, in comparison to placebo, PDE5 inhibitors significantly improved all primary endpoints in patients coping with this side effect of surgical intervention for prostate cancer. Since, under conditions of sexual stimulation, the pro-erectile effects of PDE5 inhibitors are dependent upon the unimpaired function of cavernous nerves (releasing the gaseous neurotransmitter NO) innervating the vascular and non-vascular penile smooth musculature, the treatment effect is mediocre to poor in patients who had undergone non-nerve sparing pelvic surgeries [15]. This is well in accordance with the results from a recent meta-analysis indicating that the placebo effect shows a tendency to be much more pronounced among subjects with ED associated with post-traumatic stress disorder (for example, following pelvic/prostatectomy surgery) with almost no significant differences between the treatment effects exerted by placebo and PDE5 inhibitors [16]. To date, due to the fact that a vast majority of patients require a permanent solution to overcome their ED and also in an effort to abandon the stepwise approach of adjusting the treatment regimen, an increasing number of men, following comprehensive information about the various treatment options (PDE5 inhibitors, intracavernosal injections, intraurethral/topical alprostadil) are given the opportunity of choosing the treatment that suits them best [17]. The most common adverse events of PDE5 inhibitors—headache (10–18%), flushing (5–11%), dyspepsia (4–7%) and nasal congestion/rhinitis (1–10%)—are due to the fact that PDE5 is not exclusively distributed in the corpus cavernosum penis but also present in other tissues [11]. The safety of PDE5 inhibitors was thoroughly explored during the post-marketing surveillance. In patients with coronary arterial diseases (CAD), the degree of interactions between PDE5 inhibitors and nitrates are only modest [18,19]. Although, at the time of editing this article, no evidence-based level 1 (EBL 1) study comparing the efficacies and safety of PDE5 inhibitors is available, the current data suggest that there are no major differences in their safety profiles and that the administration of PDE5 inhibitors, regardless of the active compound selected for treatment, is efficient according to clinical terms and well tolerated [20]. Recently, orodispersible tablets (ODT)) of PDE5 inhibitors (avanafil, sildenafil, tadalafil, vardenafil) have been introduced into the market. Disintegration of these tablets does not require the intake of water. ODT formulations are rapidly absorbed, with a pharmacokinetic profile similar to that of coated tablets. The partial absorption of ODT through the oral mucosa results in a greater extent of bioavailability of the active drug compound [21]. In general, in patients who are on NO donor drugs, the application of PDE5 inhibitors is generally contraindicated. It has been shown that steroidal hormones (testosterone) are of significance with regard to the post-transcriptional processing of PDE5 protein in penile erectile tissue. Thus, in hypogonadal men presenting with ED, PDE5 inhibitors may tend to be less effective, and testosterone adjunction may improve the efficacy of PDE5 inhibitors in such patients [22].

### 2.1. Sildenafil

Sildenafil citrate (VIAGRA, Pfizer Inc., New York, NY, USA) was the first-PDE5 inhibitor approved by the health authorities of the European Community (EMA) and the US (FDA) and introduced into the treatment of ED. The efficacy and safety of sildenafil (inhibiting the hydrolysis of cyclic GMP with an IC_50_ = 7 nM) was investigated in clinical trials involving more than 6000 males with different causes of ED, including patients with diabetes mellitus and those who had undergone pelvic surgery or had a history of injury to the spinal cord. The maximum plasma concentration was observed within 1 h after a single dose (25 mg to 100 mg), the T_0.5_ of the drug amounts to 4 h, and erections were found to occur within 60 min to 80 min after administration of the drug. A review of several double-blinded, placebo-controlled studies (duration up to 12 weeks) has demonstrated that the drug (at doses of 50 mg to 100 mg) improved erectile function domain scores, according to the International Index of Erectile Function (IIEF) and the Global Efficacy Questionnaire (GEQ, including the question “Did the treatment improve your erections?”), resulting in more successful intercourse attempts. This outcome was regardless of age, severity and duration of the ED or the occurrence of comorbidities. Response rates were between 86% in patients with a psychogenic ED and 52% in patients where the neuronal or endothelial production of NO was impaired due to diabetes, pelvic surgery (prostatectomy, cystectomy, rectal cancer surgery) or injuries to the spinal cord (placebo: 14–25%) [23]. This highlights that the pharmacological action of PDE5 inhibitors requires an adequate neuronal innervation of the corpus cavernosum as well as non-compromised endothelial cells.

### 2.2. Vardenafil

Vardenafil (LEVITRA) is approximately 10 times more potent than sildenafil (IC_50_ of 0.7 nM); the drug is administered in doses of 10 mg or 20 mg and the time span to maximum plasma concentration is less than 1 h (0.7 h). The plasma half-life time of vardenafil amounts to 4–5 h, however, the time from on to off-set of drug action (responsiveness) exceeds the T_0.5_ [24]. The adverse events reported are similar to those exerted by sildenafil, whereas disturbances in visual perception (blue vision) have not been reported [25]. In randomized, placebo-controlled trials (12 weeks duration, including more than 4000 men) with organic, psychogenic, or mixed ED, 54–70% of individuals from the vardenafil treatment group reported improved erections (in terms both of ability to successfully achieve vaginal penetration and maintain the erection until completion of sexual intercourse) when compared to the placebo group (23–36%) [26]. Patients who received 20 mg vardenafil exhibited a higher level of improvement in the primary outcome parameters than did those who were allocated the 10 mg dose; however, a meta-analysis did not identify a clinically relevant difference between the 10 mg and 20 mg dose [27]. In a long-term study (two years) of the efficacy of vardenafil (10 mg or 20 mg) in 490 patients, more than 90% reported an improvement in erections and successful intercourse attempts during the treatment period. It has been demonstrated that vardenafil did not affect the ability of patients with CAD to engage in sexual activity [28]. In summary, vardenafil has been demonstrated to be efficient and safe in patients with different etiologies of ED, including “difficult-to-treat” cohorts.

### 2.3. Tadalafil

The chemical structure of tadalafil (CIALIS, marketed by Eli Lilly & Co., Indianapolis, IN, USA) is different from the scaffold of sildenafil and vardenafil and exhibits little activity against other PDEs. However, when compared to sildenafil and vardenafil, the selectivity of tadalafil for the PDE isoenzyme 11A is five-fold higher (IC_50_ ratio = 7.1) (IC_50_ ratio sildenafil = 203, IC_50_ ratio vardenafil = 346). The pharmacokinetic properties of the drug are not impaired by the intake of food or alcohol. Following sexual stimulation, the majority of patients achieved penile erection approximately 30 min after the intake of 20 mg of tadalafil. The pharmacological half-life time is 18 h. In double-blinded, placebo-controlled trials (duration 12–36 weeks) which enrolled more than 1000 individuals with a mild to moderate ED of various etiologies, tadalafil (administered in doses of 2.5 mg to 25 mg) enhanced all efficacy outcome parameters. Comorbidities included hypertension, CAD, and diabetes mellitus. Tadalafil improved erections in 40–80% of patients (placebo: 35%). Approximately 70% of intercourse attempts initiated within 30 min to 36 h after the intake of a single dose of tadalafil (20 mg) were reported to be successful [29]. The efficacy of the drug at 24 h and 36 h after dosing was verified in studies conducted in 350 men with organogenic or psychogenic ED. Of these patients, 64% reported successful intercourse at 24 h to 36 h after the intake of tadalafil. The proportion of positive responses to the key questions from the Sexual Encounter Profile (SEP) concerning the ability of the patients to successfully achieve vaginal penetration (SEP Q2) and to maintain an adequate erection until completion of intercourse (SEP Q3) was significantly higher in men who were given 20 mg tadalafil than in the placebo group, and an overall improvement of 7.3 points was registered in the erectile function domain score of the IIEF (placebo = 0.1 only) [30]. Based on the extended plasma half-life time of tadalafil, low doses (up to 5 mg) administered once-daily can maintain therapeutic concentrations of the drug throughout 24 h [30]. In contrast to vardenafil and sildenafil, no facial flushing was noted with the use of tadalafil; however, up to 5% of the patients experienced severe back pain sensations. It seems likely that this particular side effect is related to the inhibitory activity of tadalafil towards the PDE11A, known to be abundantly expressed in striated musculature [31]. Studies evaluating potential interactions between tadalafil and nitrates, such as isosorbide dinitrate or glycerol trinitrate, did not provide hints regarding relevant effects of tadalafil on the reduction in blood pressure induced by nitrates [32,33].

### 2.4. Avanafil

The highly selective PDE5 inhibitor avanafil (marketed in the US by Metuchen Pharmaceuticals, LLC, Freehold, NJ, USA, under the brand name STENDRA) was originally synthesized and investigated in in vitro studies by Tanabe Pharma Corp. (Yokohama, Japan), and developed for clinical use by VIVUS Inc. (Mountain View, CA, USA). As mentioned earlier, avanafil presents a chemical structure significantly different from the nucleobase/ribose/phosphate diester model, so that the binding of the compound to the PDE5 is independent of the spatial orientation of the molecule [12]. Avanafil is absorbed very rapidly, the average time to maximum plasma concentration is 40 min (in the fasting state) or up to 1.5 h when the drug is administered in conjunction with alcoholic beverages or high fat food, and the erectile response occurs within 30 min to 40 min. Multicenter, double-blind trials enrolling 846 males with ED of psychogenic origin (n = 200) or moderate organogenic causes (n = 646) have presented favorable results. Treatment with avanafil significantly improved the IIEF-EF score (mean changes were 4.6 and 5.4 points at 100 mg or 200 mg of avanafil, respectively, whereas the administration of placebo amounted to 2 points) and also the percentage of successful sexual intercourse attempts as assessed by means of the SEP (questions 2 and 3). At 100 mg of avanafil, SEP 2 and SEP 3 rates increased from 30% to 54% and from 8% to 34%, respectively. At 200 mg, the enhancement seen was 42% to 63% (SEP 2) and 8% to 40% (SEP 3) (placebo: 10% to 20%). Successful intercourse attempts were demonstrated >6 h post-dosing, with response rates of 60–83%. However, despite its exceptional chemical properties, the overall clinical efficacy of avanafil, based on the number of subjects developing erections sufficient for sexual intercourse, does not seem to be superior to sildenafil [34,35]. The co-administration of avanafil and glycerol trinitrate had only little impact on (systolic) blood pressure and heart rate [36]. Due to its enhanced selectivity, fast onset of drug action and favorable profile of side effects, avanafil might represent a preferable first-line treatment option for men with ED who are on nitrate use. The drug may provide couples with spontaneity in conducting intercourse activities [37].

### 2.5. Udenafil

The orally active PDE5 inhibitor udenafil (marketed under the brand name ZYDENA by Dong-A Pharmaceutical Co., Youngin, South Korea) is currently available for the treatment of ED in South Korea only. In double-blind, randomized, placebo-controlled trials conducted in men with moderate to severe ED, udenafil produced a pronounced improvement in erectile function after at least 12 weeks of treatment. The ability of the patients to maintain erections, as measured by the respective IIEF-EF domain and question 3A of the SEP, was significantly enhanced by 100 mg of udenafil. The effectiveness of udenafil can be sustained for up to 12 h after administration of a single dose, thereby allowing spontaneity in the sexual activities of the patients and their partners [38,39]. Data from animal studies conducted in male rabbits and rats have provided evidence that the drug might also be effective in treating ED caused by endothelial dysfunction secondary to diabetes mellitus or hypercholesterolemia. The administration of 0.3 to 1 mg/kg udenafil resulted in an increase in intra-cavernous pressure (ICP) following the electrical stimulation of pelvic nerves. After treatment for five months with 20 mg udenafil/kg/day, erectile responses were restored and the endothelial function improved, in terms of the relaxation of isolated segments of the aorta in response to muscarinic agonists. Interestingly, plasma levels of endothelin 1 (a peptide possessing strong vasoconstrictor properties) and dimethylarginine (ADMA), a natural, endogenous inhibitor of both the neuronal and endothelial isoform of the nitric oxide synthase (NOS), decreased during treatment with udenafil [40].

### 2.6. Mirodenafil

Mirodenafil (trade name MVIX, developed and marketed by SK Chemicals Life Science, South Korea) belongs to the PDE5 inhibitors of the third generation, such as tadalafil, avanafil and udenafil, utilized as a treatment for ED. The biochemical properties of mirodenafil are different to other PDE5 inhibitors: high affinity towards the PDE5 (IC_50_ = 0.5 nM) and high selectivity for PDE5 over other PDEs. In multi-center, double-blinded, placebo-controlled studies, mirodenafil (50 mg or 100 mg) improved all primary and secondary efficacy outcome measures: questions 3 and 4 (inquiring about the ability to attain and maintain an erection, respectively), the Global Efficacy/Assessment (GEQ) question (“Did the treatment improve your erections?”), SEP questions 2 and 3 and the Life Satisfaction Checklist. Furthermore, recent evidence has been presented that mirodenafil, despite its short plasma half-life time, might be another potential drug candidate for chronic dosing in the treatment of ED. The administration of mirodenafil together with a high fat meal or alcoholic beverages does not affect in a negative manner the pharmacokinetic properties of the compound. Mirodenafil has been shown to be safe and effective in men with ED of various etiologies/severities, including those patients with concomitant diabetes or who are on antihypertensive drugs [41,42].

### 2.7. Lodenafil

Recently, the orally active PDE5 inhibitor lodenafil carbonate (developed and marketed under the brand name HELLEVA by Cristalia Produtos Quimicos Farmaceuticos, Sao Paulo, Brazil) has been introduced into clinical use as a first line treatment for ED. In isolated rabbit and human penile erectile tissue, the drug amplified the NO-dependent relaxation evoked by means of acetylcholine or transmural electrical field stimulation (EFS). In crude extracts prepared from human blood platelets, lodenafil was approximately two times more potent than sildenafil in causing inhibition of the hydrolysis of cyclic GMP mediated by the activity of PDE5 [43]. In a phase 3 clinical trial that empaneled 350 men with ED of all degrees, lodenafil (40 mg or 80 mg) significantly improved all outcome scores of the IIEF and SEP (Q2 and Q3). The adverse reactions noted—headache, flushing, rhinitis, dyspepsia, disturbances in color vision—were of only mild degree. The drug was very well tolerated and withdrawal from treatment as a result of adverse effects occurred only in a few cases. Future studies will show whether lodenafil is also effective in “difficult-to-treat” populations, such as patients with diabetes mellitus, regardless of the level of glycemic control, and in patients after nerve-sparing radical prostatectomy, and can, thus, be considered a reasonable alternative drug for the pharmacotherapy of ED [44,45].

## 3. Intra-Cavernous, Transurethral or Topical Administration of Vasoactive Drugs: Alprostadil

Aside from diseases or degenerative deteriorations of the cardiovascular system, peripheral neuropathies, as caused by diabetes mellitus or as a result of pelvic surgery (for example, radical prostatectomy or cystectomy, rectal cancer surgery), resulting in nerve damage and a deficiency in the production of neurotransmitters in pelvic nerves innervating the penis, can also significantly impair erectile function. Depending on the surgical procedure (retropubic, uni- or bilateral nerve sparing, laparoscopic/robotic techniques) the overall rate of ED in patients following radical prostatectomy/cystectomy has been determined to be 40–75% [46,47,48]. Among diabetics, the prevalence of ED ranges from 35% to almost 90% [49,50]. According to the *Cologne Male Survey*, patients with diabetes mellitus have an approximately four-fold higher risk for ED when compared to healthy males [51]. Since the pro-erectile effect of PDE5 inhibitors require an unimpaired release of NO from neuronal sources (cavernous nerves) upon sexual stimulation, these drugs tend to be less effective or even non-effective in diabetic individuals or patients following (non-nerve sparing) pelvic surgeries. Hence, alternative pharmacological treatment options utilizing drug compounds other than PDE5 inhibitors, administered locally via non-oral routes might be offered to these patients in order to target their ED effectively and safely. The safety and efficacy of intracavernosal alprostadil (marketed as CAVERJECT by Pfizer Inc., New York, NY, USA, and under the brand name EDEX by Endo Pharmaceuticals, Malvern, PA, USA), a synthetic vasodilator chemically identical to the naturally occurring prostaglandin E1 known to induce vasodilation through the activation of cyclic AMP-dependent pathways, has been evaluated as a salvage therapy for the treatment of ED in at-home study settings involving several hundreds of men with diabetes (type I or II). A satisfactory erectile response was achieved with 99% of injections of a median dose of 20 µg alprostadil (range 2.5 µg–60 µg), approximately 77% of the injections resulted in satisfactory sexual activity/intercourse, as assessed by the patients and their partners. In general, the self-regimen treatment was well tolerated, with penile pain being the most frequent adverse event reported (24%). Prolonged erection occurred in 7%, penile fibrosis in only 3% of the patients, while priapism events were only seldomly noted [52]. In a cohort of patients with ED following radical prostatectomy, 68% (of 102 men) achieved and maintained erections sufficient for sexual intercourse and 48% of patients continued long-term therapy with a mean duration of four years. The mean Sexual Health Inventory of Men (SHIM) (pre-surgery: 21.8) decreased after surgery (4.2) and increased during treatment (19.5). In the SHIM analysis, no significant difference was seen between the nerve sparing and the non-nerve sparing groups. However, 52% of the patients discontinued the intracavernosal therapy [53]. The data suggest that alprostadil injections are a safe and effective salvage option in patients who failed to show beneficial results with oral therapy. The key point for maintenance of treatment is the adjustment of the therapeutic method and titration of the dose to an optimal level to ensure satisfactory erections.

Intraurethral alprostadil is delivered through an application device called the Medicated Urethral System for Erection (MUSE) (marketed by Meda Pharma, Brussels, Belgium), containing a single-use pellet of alprostadil suspended in polyethylene glycol. The intraurethral dose recommended for treatment is 500 μg. Data from clinical studies of intraurethral alprostadil in patients with ED (aged 46–73 years), for whom therapy with PDE5 inhibitors had failed or was contraindicated, showed that it has a fast on-set of action and a favorable safety profile, with no occurrences of priapism or penile fibrosis (as seen with the intracavernosal injection). Vaginal burning or itching was sometimes reported by the female partners. However, only 35% of the patients were initially successfully treated with the 500 μg dose, and almost all of them were subjected to the 1 mg dose. After six months of at-home treatment, 43% of these patients continued to use MUSE, 33% said it was effective on every third administration only. The remaining 57% stopped taking MUSE because of the lack of effectiveness or due to side effects, such as uncomfortable irritation sensations at the site of application or urethral bleeding [54]. In conclusion, MUSE is only moderately effective in treating ED and, owing to the high drop-out rate, is not associated with high preference and acceptance rates among patients. However, in some patients not adequately responding to PDE5 inhibitors or in whom orally active drugs are contraindicated, MUSE might represent an alternative option. Due to its ease of administration, it provides patients with a higher quality of life versus the intracavernosal self-injection regimen [55,56]. In addition, intraurethral alprostadil in combination with the PDE5 inhibitor sildenafil has been evaluated as a salvage therapy for patients who failed to respond satisfactorily to either drug. The effectiveness of combination therapy was evaluated in small cohorts of patients (n = 28 or 65) with ED of different etiologies, who were dissatisfied with either oral sildenafil (100 mg) or intraurethral alprostadil (1 mg). During the combination therapy, the majority of patients reported erections sufficient for vaginal penetration, some patients reduced the dose of sildenafil citrate from 100 mg to 50 mg. Post treatment, the IIEF scores were significantly greater in the combination group (23, baseline: 10.8) when compared with either monotherapy (sildenafil: 19, alprostadil: 15). At 14 to 30 months, the combination therapy was still favored by the patients and used regularly [57,58]. Combining MUSE and sildenafil may be more efficacious in the salvage of patients who were in favor of non-invasive therapy when single-treatment modalities failed. Topical delivery of an alprostadil cream (developed by FERRING Pharmaceuticals, Kiel, Germany, and marketed under the brand name VITAROS) has been approved and is available in Canada and the EC. The major challenge with topical therapy is to ensure penetration of an effective amount of the active drug through fascial layers of the penile skin and the collagenous structure of the tunica albuginea into the penile erectile tissue (corpus cavernosum). VITAROS offers the combination of the active drug with a skin penetration enhancer, namely dodecyl-2-N,N-dimethylamino propionate (DDAIP), that improves local absorption of the active compound at the site of administration. The rationale for topical delivery is based on the potential to administer a vasoactive substance without the (potentially) systemic adverse effects of oral therapy and the invasive, uncomfortable nature of the intracavernosal or intraurethral route. After administration to the meatus and glans of the penis, alprostadil is rapidly absorbed through collateral vessels into the corpus spongiosum and corpora cavernosa, and the on-set of erection is likely to occur within 5–30 min. The duration of drug action lasts from 1 h to 2 h after dosing. The efficacy of topical alprostadil cream (100 µg, 200 µg or 300 µg of alprostadil) was investigated in multicenter, open-label, long-term studies in more than 1500 subjects with ED of various causes (21% diabetes, 44% hypertension, 12% post radical prostatectomy, 21% coronary artery disease, 16% on nitrate medication). Patients were subjected to topical alprostadil cream of either 100 µg (n = 434), 200 µg (n = 430) or 300 µg (n = 434) (placebo =434). After a four-week period, 73% of the patients increased the dose to 300 µg while 11% continued the 200 µg dose. After nine months, the majority of patients (74%), especially those who had adjusted the dose to 300 µg, demonstrated an overall improvement in erectile function as assessed by means of the IIEF domain score (*p* < 0.001). Significant increases were also observed in all secondary outcome variables (vaginal penetration, maintenance of erection until ejaculation, and Life Satisfaction Checklist). The most common adverse events were limited to the application site and involved burning or erythema (12%) and pain sensations (4%). Only a very few patients (1.3%) reported prolonged or painful erections following application of the drug. Vaginal burning or itching (2.1%) was reported by a number of partners [59,60]. A randomized cross-over clinical study showed that the delivery of the drug within the urethral meatus instead of applying it to the tip of the penis can increase the level of treatment efficacy without increasing the incidence of adverse events [61].

**Figure 1:**
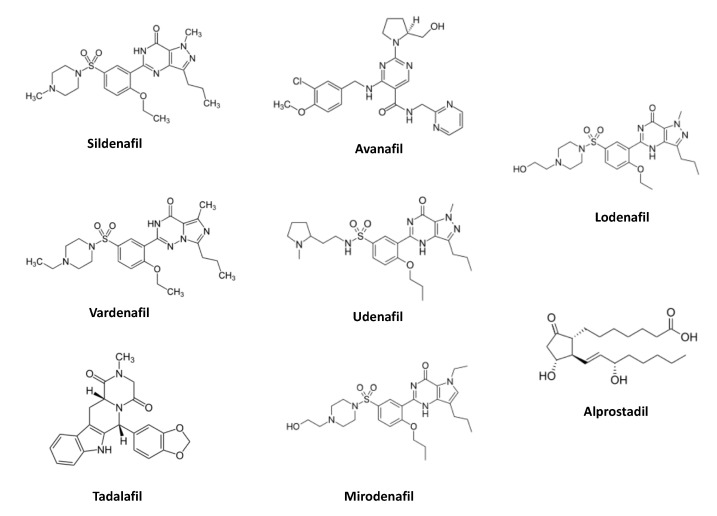
Structural formulas of the vasoactive drugs - phosphodiesterase (PDE)5 inhibitors and alprostadil, a derivative of the natural occurring compound prostaglandin E1)—commonly administered in the pharmacotherapy of male erectile dysfunction (ED). The PDE5 inhibitors share three major domains: a guanine-like purine system, a sugar (ribose)-/phenyl- and a sulphonamide-like entity. Note that the chemical structure of avanafil is significantly different from the standard model.

See Figure 1 for the chemical structures of the current selective PDE5 inhibitors and alprostadil, and Table 1 for an overview on the vasoactive drug formulations (for oral or local application) most commonly used in the treatment of male ED.

## 4. Conclusions

Pharmacological intervention into intracellular pathways regulating smooth muscle tone has become a common strategy in urology for relief of symptoms caused by dysfunctions of the urogenital tract. Based on the physiological mechanisms regulating the male genital tract, vasoactive drugs (such as selective PDE5 inhibitors or prostaglandin E1) modulating signal transduction cascades represent a straightforward logical approach for effectively treating ED of various origins (vasculogenic/neurogenic/psychogenic) in a significant number of patients [62]. While some strategies of treatment target the cyclic GMP system, other approaches take into account the cyclic AMP system. The combination of active agents in order to affect multiple intracellular targets is also being considered. In order to ensure a pharmacological effect without significant adverse events, especially on the cardiovascular system, drug action should be limited to the target tissue (the erectile vascular and non-vascular smooth musculature). This can be contracted either by a certain degree of tissue selectivity of orally available drugs or the local application of active compounds via suitable routes [63,64]. An improved bioavailability, a faster on-set and sustained duration of drug action, in combination with a high response rate and the advantage of an on-demand application, might be associated with more spontaneous sexual activity for ED patients. The development of first-line oral treatments demonstrating advanced efficacy over the previous options will remain an important topic.

## Figures and Tables

**Table 1:** Clinical trials (randomized, placebo-controlled and double-blinded) or integrated analyses of studies that have investigated the efficacy and safety of vasoactive drugs in cohorts of patients with ED of organogenic/neurogenic origin. All studies listed meet the evidence-based medicine (EBM) level 1b.

Drug Compound	Key Clinical Studies/Integrated Analysis	Key Pharmacological Data	Route of Administration	Product Availability
Sildenafil	Carson, CC; et al. *UROLOGY* **2002**, 60 (Suppl 2): 12–27 [23]	IC_50_ = 7 nM T_max_ (area under the time-concentration curve) = 60 min (after a single dose of 50 mg or 100 mg) T (lambda)_0.5_ = 4 h	orally active (PDE5 inhibitor)	The Americas, EC, Africa, Asia(-Pacific), Australia
Vardenafil	Stief, CG; et al. *Int J Clin Pract* **2004**, 58: 230–239 [26] Brock, GB; et al. *J Urol* **2002**, 168: 1332–1336 [28]	IC_50_ = 0.7 nM T_max_ = 0.7 to 1 h (after a single dose of 10 mg or 20 mg) T_0.5_ = 4 to 5 h	orally active (PDE5 inhibitor)	The Americas, EC, Africa, Asia(-Pacific), Australia
Tadalafil	Porst, H; et al. *UROLOGY* **2003**, 62: 121–125 [29]	IC_50_ = 5 nM T_max_ = 0.8 to 1.2 h (after a single dose of 20 mg) T_0.5_ = 18 h	orally active (PDE5 inhibitor)	The Americas, EC, Africa, Asia(-Pacific), Australia
Avanafill	Mulhall, JP; et al. *J Urol* **2013**, 189: 2229–2236 [13] Zhao, C; et al. *BJU Int* **2012**, 110: 1801–1806 [34] Goldstein, I; et al. *J Sex Med* **2012**, 9: 1122–1133 [35]	IC_50_ = 5.2 nM T_max_ = 30 to 40 min (after a single dose of 100 mg or 200 mg T_0.5_ = 6 h	orally active (PDE5 inhibitor)	The Americas, EC, Asia(-Pacific)
Udenafil	Zhao, C; et al. *Eur Urol* **2011**, 60: 380–387 [38] Park, HJ; et al. *J Sex Med* **2010**, 7: 2209–2216 [39]	IC_50_ = 8.2 nM T_max_ = 0.8 to 1 h (after a single dose of 100 mg or 200 mg) T_0.5_ = 7 to 12 h	orally active (PDE5 inhibitor)	Asia(-Pacific (South Korea only)
Mirodenafil	Paick, JS; et al. *J Sex Med* **2008**, 5: 2672–2680 [41] Cho, MC; Paick, JS. *Ther Adv Urol* **2016**, 8: 100–117 [42]	IC_50_ = 0.5 nM T_max_ = 60 min (after a single dose of 50 mg or 100 mg) T_0.5_ = 4 to 5 h	orally active (PDE5 inhibitor)	Asia(-Pacific (South Korea only)
Lodenafil	Glina, S; et al. *J Sex Med* **2010**, 7: 1928–1936 [43] Hatzimouratidis, K; et al. *J Sex Med* **2016**, 3: 465–488 [45]	IC_50_ = 0.2 nM T_max_ = 1.5 h (after a single dose of 40 mg or 80 mg) T_0.5_ = 3.3 h	orally active (PDE5 inhibitor)	South Americ (Brazil only)
Alprostadil (chemically identical to prostaglandin E1) CAVERJECT/EDEX	Perimenis, P; et al. *Asian J Androl* **2006**, 8: 219–224 [52] Raina, R; et al. *Int J Impot Res (IJIR)* **2003**, 15: 318–322 [52]	Compound is administered locally, thus not distributed systemically (see Route of administration)	Intracavernosal route of administration (on-demand self-injection regimen)	The Americas, EC, Africa, Asia(-Pacific), Australia
Alprostadil (Prostaglandin E1) MUSE	Costabile, RA; et al. *J Urol* **1998**, 160: 1325–1328 [53] Khan, MA; et al. *Curr Med Res Opin* **2002**, 18: 64–67 [56]	Compound is administered locally, thus not distributed systemically (see Route of administration)	intra-/transurethral route of administration	The Americas, EC, Asia(-Pacific), Australia
Alprostadil (Prostaglandin E1) VITAROS	Rooney, M; et al. *J Sex Med* **2009**, 6: 520–534 [59] Padma-Nathan, H; Yeager, JL. *UROLOGY* **2006**, 68: 386–391 [60]	Compound is administered locally, thus not distributed systemically (see Route of administration)	topical/transdermal route of administration	The Americas (Canada), EC
